# Decoy Receptor 3 Improves Survival in Experimental Sepsis by Suppressing the Inflammatory Response and Lymphocyte Apoptosis

**DOI:** 10.1371/journal.pone.0131680

**Published:** 2015-06-29

**Authors:** DongYu Liang, YanQiang Hou, XiaoLi Lou, HongWei Chen

**Affiliations:** Department of Central Laboratory, Songjiang Hospital Affiliated First People’s Hospital, Shanghai Jiao Tong University, Shanghai, China; Chinese Academy of Sciences, CHINA

## Abstract

**Purpose:**

Unbalanced inflammatory response and lymphocyte apoptosis is associated with high mortality in septic patients. Decoy receptor 3 (DcR3), a member of the tumor necrosis factor receptor superfamily, is an anti-inflammatory and anti-apoptotic factor. Recently, DcR3 expression was found to be increased in septic patients. This study evaluated the therapeutic effect and mechanisms of DcR3 on cecal ligation and puncture (CLP)-induced sepsis in mice.

**Methods:**

C57BL/6 mice were subjected to CLP-induced polymicrobial sepsis. DcR3 Fc was intravenously injected 30 min before and 6 h after CLP. Bacterial clearance, cytokine production, histology, lymphocyte apoptosis and survival were evaluated. Furthermore, we investigated the systemic effects of DcR3 in *in vitro* lymphocyte apoptosis regulation.

**Results:**

Our results demonstrated that DcR3 protein treatments significantly improved survival in septic mice (*p* <0.05). Treatment with DcR3 protein significantly reduced the inflammatory response and decreased lymphocyte apoptosis in the thymus and spleen. Histopathological findings of the lung and liver showed milder impairment after DcR3 administration. *In vitro* experiments showed that DcR3 Fc inhibited Fas-FasL mediated lymphocyte apoptosis.

**Conclusions:**

Treatment with the DcR3 protein protects mice from sepsis by suppressing the inflammatory response and lymphocyte apoptosis. DcR3 protein may be useful in treatment of sepsis.

## Introduction

Despite advances in supportive care, sepsis remains one of the most challenging clinical problems due to its high morbidity and mortality in children and adults [1, 2]. The pathophysiological process of sepsis is a complex immunologic response with pro-inflammatory and anti-inflammatory mechanisms alternatively predominating [3]. It has been hypothesized that if death occurs in the first few days, it is most likely due to the exaggerated inflammatory response [4]. Recent studies have found that most patients survive the initial pro-inflammatory state but tend to die during the immunosuppression period [5]. Temporary immunosuppression during sepsis appears to be closely correlated with mortality and secondary infection [6]. Accumulating evidence suggests that immune effector cell apoptosis in sepsis is associated with the development of the immunosuppression [7, 8]. Apoptotic cell uptake by phagocytic cells such as macrophages and dendritic cells (DCs) leads to an immunosuppressive state by inducing the production of anti-inflammatory cytokines and suppressing the release of pro-inflammatory cytokines [9, 10]. Thus, new drugs with effective anti-inflammatory profiles as well as immunomodulatory properties would be promising and valuable.

Expression of some co-stimulatory/inhibitory molecules is markedly altered in sepsis. These molecules appear to be associated with morbidity and mortality in septic models as well as patients with sepsis [11, 12]. Decoy receptor 3 (DcR3, also known as TR6) is a newly identified decoy receptor. It is a secreted protein that belongs to the tumor necrosis factor (TNF) receptor family. DcR3 has three primary ligands: FasL, LIGHT, and TL1A. DcR3 can bind FasL to protect against FasL-mediated apoptosis of lymphocytes and several tumor cell types [13–15]. DcR3 can also bind LIGHT and inhibit LIGHT-induced apoptosis [16]. DcR3 can induce T cell activation via binding to TL1A [17].

We recently found that DcR3 expression is elevated in patients with sepsis and is related to sepsis mortality [18]. These findings suggested that DcR3 might be implicated in the pathogenicity of sepsis.

In this study, we investigated the effect of DcR3 on survival in a murine cecal ligation and puncture (CLP) model of sepsis. In addition, we attempted to elucidate the potential mechanisms underlying the putative beneficial effect.

## Materials and Methods

### Mice preparation

Adult C57BL/6 mice (8–10 weeks age), with a body weight of 22–30 g, were obtained from the Animal Center of the First People’s Hospital Affiliated with Shanghai Jiaotong University China. They were maintained in a specific pathogen-free facility at a temperature of 22 ±2°C with 12 h light and dark cycles and 50% relative humidity. All procedures were approved by the Committee on the Ethics of Medical Scientific Research of the First People’s Hospital, Shanghai Jiaotong University (Permit Number: 2012KY041). All treatments were humane and in accordance with the guidelines for the Care and Use of Laboratory Animals of the National Institutes of Health.

### Cecal ligation and puncture mouse model

The sepsis model was induced as previously described [19]. Briefly, mice were anesthetized with isoflurane and a 1- to 2-cm midline incision was made after disinfecting the abdomen. The cecum was exposed and ligated with 3–0 silk tied 1 cm from the tip, and was subjected to a double puncture with a 20-gauge needle. The bowel was returned to its original position, and the incision was closed in dual layers. Sham mice had the peritoneum opened and the bowel exposed, but without ligation and puncture. After surgery, all mice received 1 ml sterile physiologic saline solution. To minimize suffering, all mice were anesthetized with isoflurane prior to surgery and subsequent sacrifice.

For the survival study, we used the death as the clinical endpoint. All mice were monitored every 6 h for 7 days and euthanized when they were found in a moribund state as identified by labored breathing and/or non-responsiveness to cage tapping. Moribund mice were anesthetized with ketamine prior to cervical dislocation. At the end of the experiment, all surviving mice were euthanized with ketamine followed by cervical dislocation.

### Experimental protocol

DcR3 Fc and Ig Fc were purchased from Sigma (Saint Louis, MO, USA). Briefly, DcR3 Fc was produced from a DNA sequence encoding the signal peptide from human CD33 joined with amino acid residues 24–300 of human DcR3 which was fused to the Fc region of human Ig G1 by a polypeptide linker. The recombinant DcR3/Fc chimera was measured by its ability to inhibit the biological activity of Fas-ligand-induced apoptosis of Jurkat cells. Forty mice were randomly divided into four groups (ten mice per group): sham group, CLP group, CLP plus Ig Fc group, and CLP plus DcR3 Fc group. DcR3 Fc was dissolved in sterile distilled saline and 100 μg DcR3 Fc was intravenously injected in mice 30 min before CLP and 6 h after CLP. For the sham and CLP groups, mice received an equal volume of saline. For control purposes, the CLP plus Ig Fc group received an equal amount of Ig Fc. Twenty-four hours after surgery, blood, lung, liver, spleen and thymus were collected. Peritoneal lavage fluid was collected by injecting 2 ml phosphate buffered saline (PBS) into the peritoneal cavity. Another forty mice were observed for the survival of animals over the subsequent 7 d.

### Histological examinations

Tissue samples, including lung and liver, were collected 24 h after surgery and immediately fixed in 4% paraformaldehyde. After 12 or 24 h, tissue sections were embedded in paraffin, cut into 4–5 μm sections, and stained with hematoxylin–eosin. Two experienced pathologists who were blinded to the protocol performed the histological examinations. To grade the degree of lung injury, a scoring system was used based on the following histological features: edema, hyperemia and congestion, neutrophil margination and tissue infiltration, intra-alveolar hemorrhage and debris, and cellular hyperplasia. Each feature was graded as absent, mild, moderate, or severe, with a score of 0 to 3, and total scores were calculated for each animal [20]. The scoring system for evaluating the degree of liver injury was based on the scale of cell necrosis: 0, none; 1, individual cell necrosis; 2, up to 30% lobular necrosis; 3, up to 60% lobular necrosis; and 4, more than 60% lobular necrosis, and total scores were calculated for each animal.

### Cytokine determination

Blood and peritoneal lavage fluid were harvested from mice 24 h after surgery and centrifuged for 10 min at 300 g. The levels of TNF-α, IL-6, and IFN-γ were quantified using a murine ELISA kit (Bender Systems, USA) according to the manufacturer’s instructions.

### Measurements of apoptosis in thymus and spleen

Thymus and spleen tissues were harvested from mice 24 h after surgery. Single-cell suspensions from thymus were prepared. CD3+ T cells were stained using fluorochrome-conjugated annexin V and propidium iodide (PI), and analyzed using a FASCalibur apparatus and CellQuest software (Becton Dickinson, USA). Annexin V-positive cells were considered apoptotic cells. Terminal deoxynucleotidyl transferase- mediated dUTP nick-end labeling (TUNEL) assays were performed using the ApopTag Plus Peroxidase *In Situ* Apoptosis Detection Kit (Chemi-Con, USA) according to the manufacturer’s instructions. Briefly, after fixation with 4% paraformaldehyde, spleen samples were incubated for 10 min in equilibration buffer. The TUNEL reaction mixture was added and sections were incubated for 1 h in a 37°C humidified chamber. After stopping the reaction, sections were visualized using the anti-digoxigenin-peroxidase antibody and DAB, and counterstained with bis-benzamide. Positive cells were identified and counted using light microscopy. The percentage of TUNEL-positive cells was used to determine the apoptosis rate. At least 1000 cells per section were examined in five randomly selected fields (400×). At least four sections were used to calculate the apoptosis rate of each group.

### Determination of lymphocyte counts in blood, spleen, and thymus

Blood, spleen, and thymus were collected 24 h after CLP. For spleen and thymus, single-cell suspensions were first prepared. After erythrocyte lysis, the total cell number was recorded. Lymphocyte subgroups were also stained with fluorochrome-conjugated antibodies against cell subset-specific surface markers. Lymphocyte counts were calculated based on the total cell count and lymphocyte subgroup percentage obtained by FACS analyses.

### DcR3 Fc administration and FasL-induced lymphocyte apoptosis

Using lymphocyte separation media, lymphocytes were separated from whole blood from septic persons and randomly divided into four groups: normal, FasL, FasL plus Ig Fc, and FasL plus DcR3 Fc. Cells were pretreated for 1 h with 10 ng/ml DcR3 Fc or 10 ng/ml Ig Fc, followed by incubation for an additional 12 h with 100 ng/ml recombinant human FasL (rHuFasL; R&D Systems). Cells were then collected and apoptosis was detected using annexin V and PI double staining and western blot analyses.

### Western blot assay

Cells were lysed with raidoimmunoprecipitation assay (RIPA) reagent (Beyotime, ShangHai, China) in the presence of protease inhibitors (Sigma, USA) and total protein quantified with the bicinchoninic acid (BCA) protein assay kit (Beyotime, Shanghai, China). A total of 20 μg of protein were separated by sodium dodecyl sulfate-polyacrylamide gel electrophoresis (SDS–PAGE) and electro-transferred onto a polyvinylidene fluoride (PVDF) membrane (BioRad, Hercules, CA, USA). Expression of cleaved caspase 8 was detected using mouse anti-human caspase 8 monoclonal antibody (Cell Signaling Technology, Beverly, MA) and HRP-conjugated sheep anti-mouse antibody (Amersham Bioscience). Expression of cleaved PARP was detected using rabbit anti-human PARP polyclonal antibody (Cell Signaling Technology) and horseradish peroxidase (HRP)-conjugated sheep anti-rabbit antibody (Amersham Bioscience). Expression of GAPDH was detected using rabbit anti-human GAPDH polyclonal antibody (Amersham Bioscience) and HRP-conjugated sheep anti-rabbit antibody (Amersham Bioscience). Protein expression was visualized using ECL Plus reagent (Amersham Bioscience) and a Chemilumino analyzer GelDoc XR system (Biorad, USA).

### Statistical analyses

SPSS 19.0 statistical software was used for all analyses. Data are expressed as the mean ± standard deviation (SD). Kaplan–Meier plots were used to describe survival data and a log-rank test was performed to assess the statistical differences. For multiple comparisons, one-way analyses of variance (ANOVA) were used to compare differences between groups. A value of *p*<0.05 was considered statistically significant.

## Results

### Effect of DcR3 on survival of mice with sepsis

**Fig 1:**
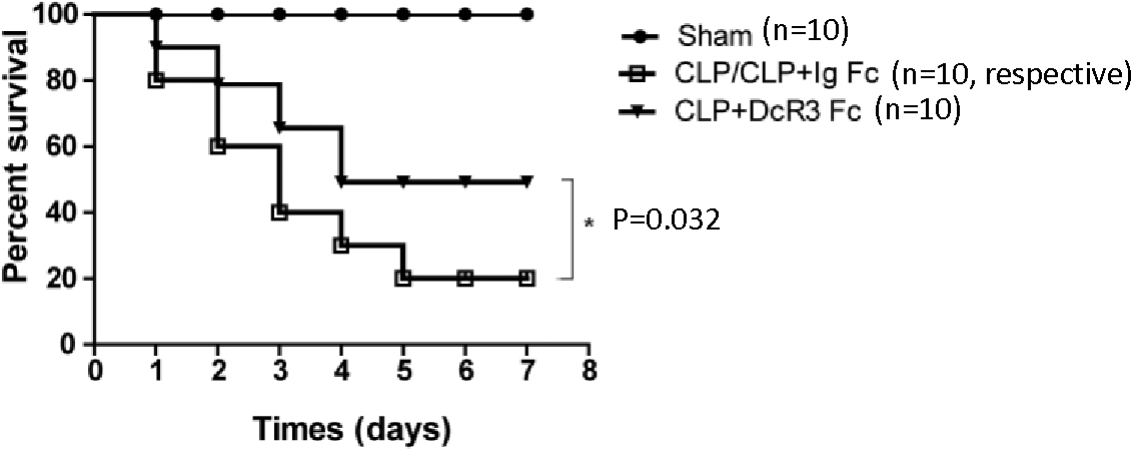
DcR3 Fc administration protects mice from CLP-induced lethality. Mice were randomly divided into four groups (ten mice/group): sham, CLP, CLP plus Ig Fc, and CLP plus DcR3 Fc. For the CLP plus DcR3 Fc group, 100 μg DcR3 Fc was intravenously infused 30 min before and 6 h after CLP. For CLP plus Ig Fc, 100 μg Ig Fc was intravenously infused 30 min before and 6 h after CLP. Other groups received the same volume of saline as a control. Survival was monitored for 7 d. Survival rates were analyzed using log-rank tests. *p <0.05.

In order to investigate whether DcR3 was beneficial for CLP mice, survival were observed for 7 d after surgery. All mice in the sham group survived during this period, whereas in the CLP and CLP plus Ig Fc group, the survival rate was only 20%. In contrast, mice treated with DcR3 Fc showed a significantly higher survival rate compared with the CLP and CLP plus Ig Fc groups (50% vs. 20%, *p*<0.05) (Fig 1). The administration of DcR3 Fc significantly improved survival rate during the study period. The results indicate that administration of DcR3 Fc protects mice against sepsis induced by CLP.

### DcR3 ameliorated liver and lung injury

**Fig 2:**
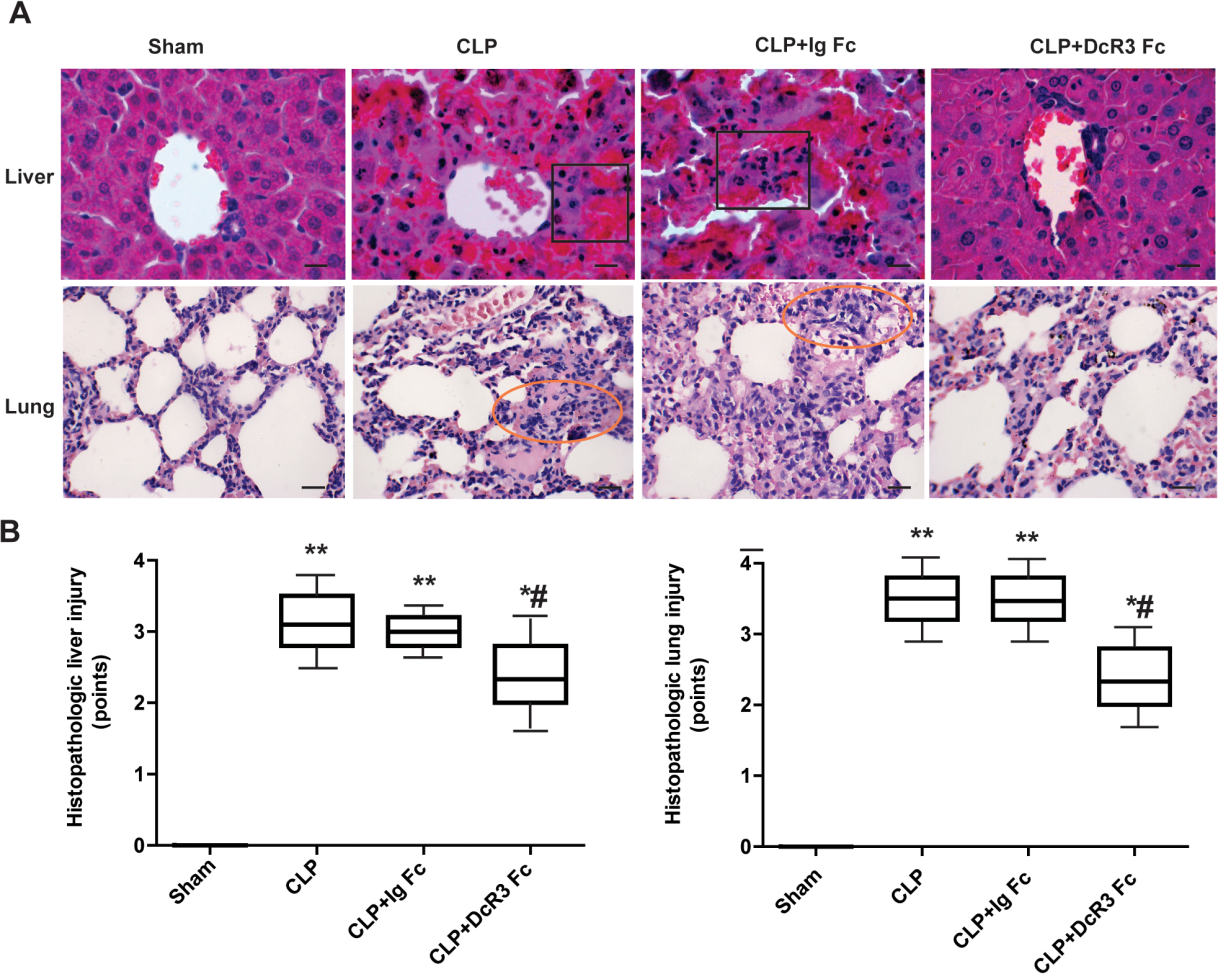
Histopathological changes in septic mice after DcR3 Fc administration. Liver and lung tissues were harvested 24 h after CLP. Hematoxylin–eosin staining was performed to examine histopathological changes (400 ×). (A) Representative images for each group. Lung injury was characterized by thickening of the alveolar wall, accumulation of neutrophils into the interstitium and impaired alveoli in CLP mice (circle areas). Liver injury was evidenced by swollen hepatocytes, inflammatory infiltration and hemorrhagic necrosis in CLP mice (square areas). DcR3 Fc treatment protected mice against sepsis-induced liver and lung damage. Lung displayed less alveolar destruction and alveolar epithelial hyperplasia when treated with DcR3 Fc. Hepatocytes showed less ballooning lesions. (B) The severity of lung and liver injury was scored as described above. Scale bars in right lower corner represent 25μm. **p <0.01, *p <0.05 vs. sham; #*p* <0.05 vs. CLP.

To investigate whether DcR3 Fc administration is associated with pathological changes in lung and liver tissue, Hematoxylin–eosin staining was performed. Lung injury, accompanied by infiltration of inflammatory cells into the lung interstitium and alveolar space, alveolar wall thickening, consolidation, and alveolar hemorrhage, was noted in the CLP and CLP plus Ig Fc groups. Liver injury was evidenced by swollen hepatocytes and absent hepatic sinusoidin the CLP and CLP plus Ig Fc groups. DcR3 Fc treatment significantly prevented these pathological changes (Fig 2). These results demonstrate that DcR3 Fc treatment significantly improves the liver and lung histopathology in CLP-induced septic mice.

### DcR3 decreased cytokine production

**Fig 3:**
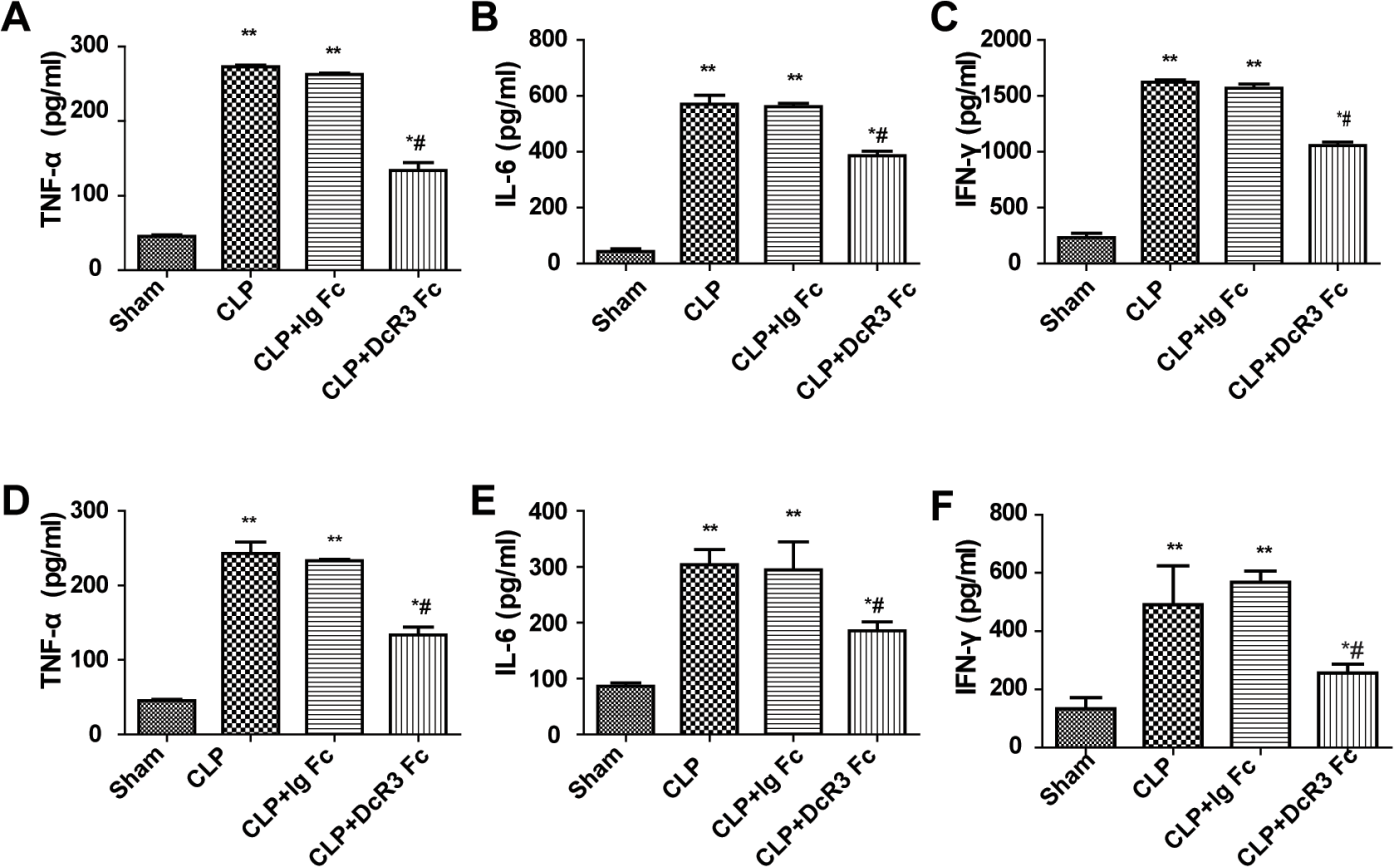
DcR3 Fc administration reduced inflammatory cytokine release in blood and peritoneal lavage fluid. Blood and peritoneal lavage fluid were collected 24 h after CLP. ELISA was performed to measure cytokine levels. DcR3 Fc administration reduced TNF-α, IL-6, IFN-γ secretion. (A–C) Representative levels of pro-inflammatory cytokines in blood. (D–E) Representative levels of pro-inflammatory cytokines in peritoneal lavage fluid (n = 6 mice/group). **p <0.01, *p <0.05 vs. sham; # *p* <0.05 vs. CLP.

To assess the effects of DcR3 Fc administration on cytokine production during the CLP model of sepsis, the levels of TNF-α, IL-6, and IFN-γ in the blood and peritoneal lavage fluid were analyzed. Twenty-four hours after surgery, the levels of TNF-α, IL-6, and IFN-γ were significantly higher in the blood and peritoneal lavage fluid of the CLP group compared with the sham group. Administration with DcR3 Fc significantly attenuated inflammatory cytokine production in the CLP group (Fig 3). These results suggest that DcR3 Fc treatment downregulates cytokines in the blood and peritoneal lavage fluid of CLP-challenged mice.

### DcR3 inhibited lymphocyte apoptosis in the spleen and thymus of septic mice

**Fig 4:**
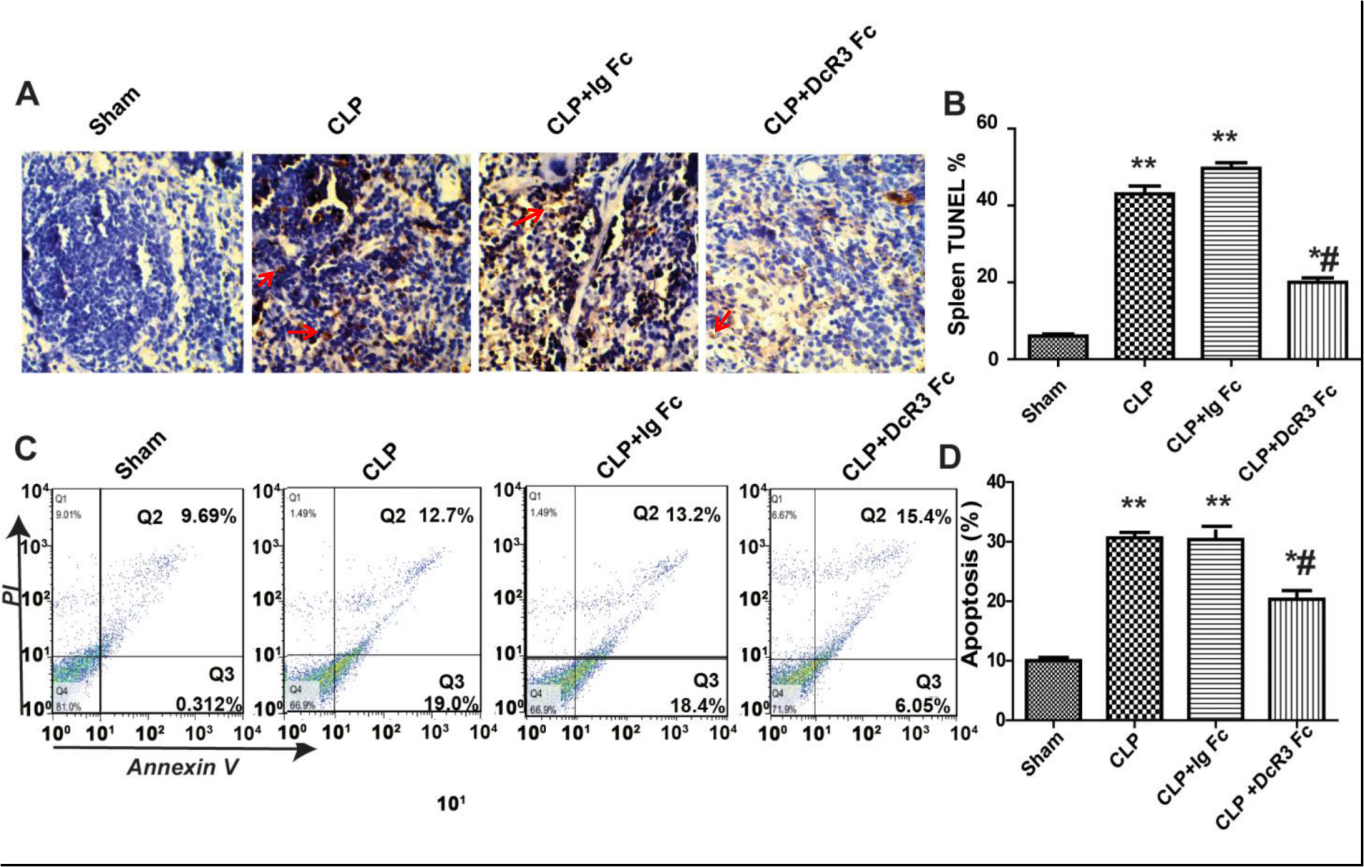
DcR3 Fc administration inhibits apoptosis in the spleen and thymus. Spleen and thymus were harvested 24 h after CLP. A. Representative sections analyzed using *in situ* TUNEL assays in spleen. Apoptotic cells are stained with a yellow-brown color in the nuclei (red arrows). B. The percentage of TUNEL-positive cells represents apoptotic cells in spleen. C. Flow cytometry dot plots of annexin V/PI staining. D. The percentage of FACS-positive cells represents apoptotic cells in thymus. Apoptosis was calculated as the percentage of annexin V-positive cells that were the sum of the percentage of cells included in the lower and upper right quadrants. Bars represent the mean ±SD (n = 6 each group). **p <0.01, *p <0.05 vs. sham; # *p* <0.05 vs. CLP.

To investigate whether DcR3 Fc administration is associated with a reduction of lymphocyte apoptosis, TUNEL staining and flow cytometry assays were performed. TUNEL assays showed that septic mice showed enhanced apoptosis activation in the spleen. DcR3 Fc administration effectively inhibited the depletion of splenocytes. Additionally, flow cytometry assays indicated a higher percentage of apoptotic thymus cells in CLP mice compared with the sham group (*p* <0.05) (Fig 4). DcR3 Fc administration effectively reduced the number of apoptotic lymphocytes in the thymus of septic mice (*p* <0.05) (Fig 4). These results indicate that CLP-induced septic mice display increased cell apoptosis in the spleen and thymus and that DcR3 Fc treatment significantly reduces the number of apoptotic cells.

### DcR3 increased lymphocyte numbers in peripheral blood, spleen, and thymus

**Fig 5:**
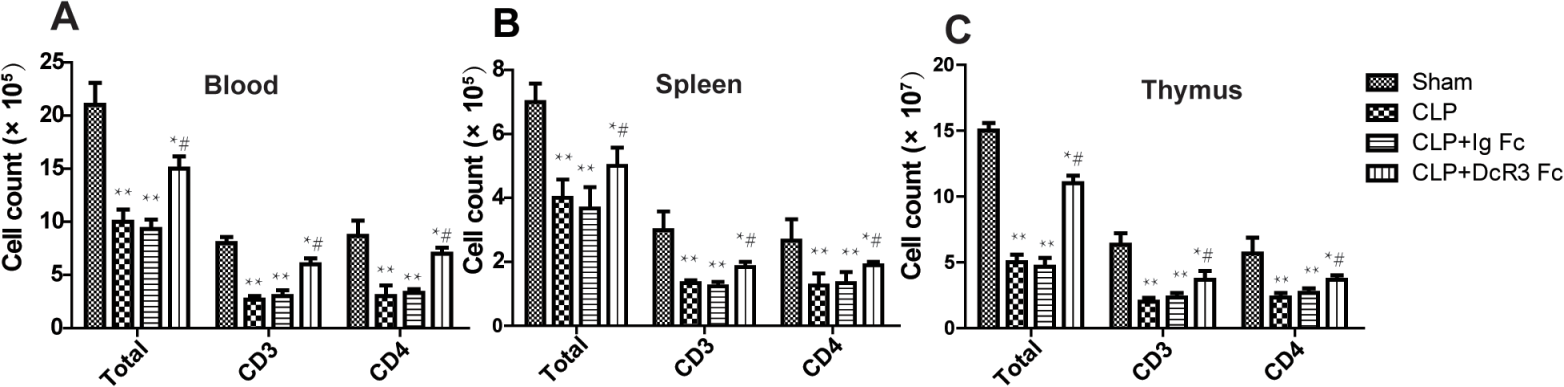
Cell numbers in blood, spleen, and thymus. Blood, thymus, and spleen were harvested 24 h after CLP. For spleen and thymus, single-cell suspensions were prepared and the total cell number was counted after erythrocyte lysis. Using FACS analyses, lymphocyte numbers were acquired by the total number and percent of lymphocyte subgroup. **p <0.01, *p <0.05 vs. sham; # *p* <0.05 vs. CLP.

To investigate the effects of DcR3 Fc administration on lymphocyte apoptosis, lymphocyte numbers in peripheral blood, spleen and thymus were detected by flow cytometry. The number of total lymphocytes was higher in the blood of the CLP plus DcR3 Fc group compared to the CLP-only or Ig Fc control groups. Similar results were observed in the spleen and thymus (Fig 5). These results suggest that DcR3 Fc treatment increases lymphocyte numbers in peripheral blood, spleen, and thymus.

### Effect of DcR3 administration on FasL-induced lymphocyte apoptosis

**Fig 6:**
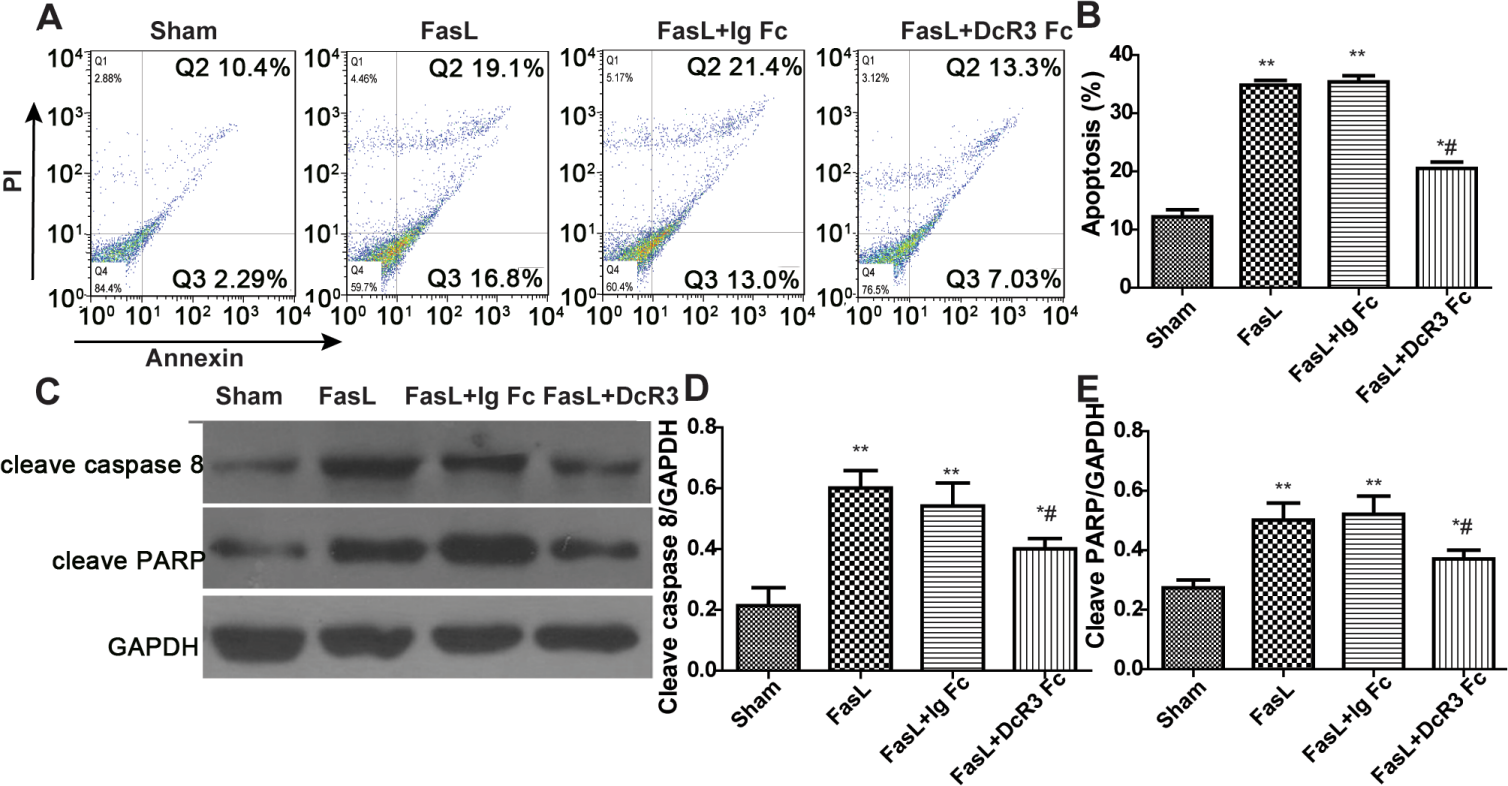
DcR3 Fc administration inhibited FasL-induced apoptosis. DcR3 Fc administration decreased human peripheral lymphocyte apoptosis induced by FasL. Peripheral lymphocytes cells were obtained from septic shock patients using Ficoll-Paque Plus and randomly divided into four groups: control, FasL-induced, FasL plus Ig Fc, and FasL plus DcR3 Fc. Cells were pretreated for 1 h with 10 ng/ml DcR3 Fc or 10 ng/ml Ig Fc, followed by the addition of 100 ng/ml FasL to induce apoptosis. After 12 h, cells were collected and apoptosis was evaluated by double staining with annexin V and propidium iodide (PI) and western blot analyses. (A) Representative micrographs of apoptosis detected by flow cytometry. (B) The percentage of FACS-positive cells represents apoptotic cells. Apoptosis was calculated as the percentage of annexin V-positive cells that were the sum of the percentage of cells included in the lower and upper right quadrants. (C) Western blot analyses of caspase 3 and cleaved poly (ADP-ribose) polymerase (PARP) levels in each group. (D–E) The relative levels of caspase 3 and cleaved PARP in each group after normalization to GAPDH. **p <0.01, *p <0.05 vs. sham; # *p* <0.05 vs. CLP.

To better understand some of the mechanisms whereby DcR3 inhibits lymphocytes apoptosis during the CLP model of sepsis, we induced apoptosis by stimulation with recombinant FasL. We found that preincubation with DcR3 Fc significantly inhibited FasL-mediated lymphocyte apoptosis. Western blot assays indicated that cleavage of caspase 8 and PARP was also inhibited by pretreatment with 10 ng/ml DcR3 Fc (Fig 6). These results indicate that DcR3 pretreatment decreases FasL-induced peripheral lymphocyte apoptosis.

## Discussion

Our previous study demonstrated that DcR3 expression was upregulated in patients with sepsis [18] and the role of increased levels of DcR3 in sepsis patients was particularly interesting. DcR3 is a decoy receptor that belongs to the tumor necrosis factor receptor superfamily (TNFRSF). Due to its lack of an apparent transmembrane domain, DcR3 is a secreted protein. DcR3 has three known ligands: FasL, LIGHT, and TL1A. DcR3 binds these ligands and inhibits their interaction with cell surface-bound receptors. Because of its role in inhibiting apoptosis via binding to these ligands, DcR3 has been extensively studied in cancer [21, 22].

DcR3 expression has also been demonstrated in various acute and chronic inflammatory conditions, including inflammatory bowel disease (IBD) and acute respiratory distress syndrome (ARDS) [23, 24]. In these studies, it was suggested that DcR3 mediated anti-inflammation by skewing the Th1/Th2 cell balance toward the Th2 response and suppressing the phagocytic activity of macrophages [25].

In this study, we found that administration DcR3 before and after CLP effectively protected mice from CLP-induced sepsis. The potential mechanisms underlining this therapeutic effect might associated with different aspects of sepsis, including reduced inflammatory responses and decreased lymphocyte apoptosis.

Sepsis is a systemic inflammatory response caused by infection. Unlike other inflammatory diseases, it is characterized by two distinct phases [3]. The early cytokine-mediated pro-inflammatory response is activated against invading pathogens. Destructive inflammatory responses are hallmark signs of sepsis. Pro-inflammatory cytokines such as TNF-α, IL-6, and IFN-γ are released in this phase, resulting in multi-organ dysfunction [26, 27]. Although the pro-inflammatory response during the early phase of sepsis is considered a normal defense, mild down-regulation of pro-inflammatory cytokines during this phase might be beneficial [28]. In agreement with previous studies which found that DcR3 inhibits the functions in the anti-inflammatory response by skewing the Th1/Th2 cell balance toward Th2 responses [25], we found that DcR3 could suppress TNF-α and IL-6 release in blood and peritoneal lavage fluid. However, other potential mechanisms in the pathophysiology of sepsis are likely, as previous studies of various anti-inflammatory agents, including TNF-α and IL-1 antagonists, showed little or no benefit in sepsis patients [29]. Due to decreased T cells and the release of anti-inflammatory cytokines, the immune system exhibits immune suppression [30]. This immune suppression phase may determine the outcome of sepsis. Moreover, results from animal models have demonstrated that altered proapoptotic or antiapoptotic protein expression improves survival during sepsis [31, 32]. T cells are essential regulators in acquired immunity. A series of studies illustrated that mice lacking T cells succumb more readily to septic insult [33]. It was reported that DcR3 might promote inflammation by inhibiting FasL-mediated apoptosis in T cells in the diseased area [34]. In present study, we found that administration of DcR3 protected immune cells in the thymus and spleen from apoptosis. Enhanced adaptive immune function may be another critical factor contributing to the therapeutic effect of DcR3 against CLP-induced sepsis.

DcR3 is suggested to inhibit apoptosis by binding to FasL and LIGHT. FasL exists on most immune cells. Increased apoptosis in response to polymicrobial sepsis and CLP has been reported, primarily in the immune cell population expressing the FasL receptor [35]. In addition, Ackerman et al. reported that organ damage and mortality associated with sepsis in CLP-induced mice is due at least in part to activation of the Fas-FasL signaling pathway [36]. To evaluate whether the DcR3 Fc protein protects against immune cell apoptosis, we performed *in vitro* overexpression experiments. Peripheral lymphocytes cells obtained from septic shock patients were incubated with FasL in the presence of DcR3 Fc or Ig Fc. Our data revealed that DcR3 pretreatment decreased FasL-induced peripheral lymphocyte apoptosis.

However, clearance of the bacterial burden from the blood and peritoneal lavage fluid of septic mice was not altered (data not shown). We suggest that the unaltered level of bacterial burden clearance may be related to the suppression of macrophage phagocytic activity by DcR3. Unfortunately, all mice were euthanized after observation for 7 days, and no further data were obtained. Moreover, there is an apparent contradiction between the assumption that DcR3 concentration is related to sepsis mortality [18], and the potentially beneficial effects of DcR3 administration in CLP mice. It is possible that in previous studies the serum samples of patients with sepsis were collected within 24 h after being admitted to the intensive care unit (ICU). As reported above, mild down-regulation of pro-inflammatory cytokines during the early phase might be beneficial for sepsis, however, inhibition of pro-inflammatory cytokine release might lead to immune suppression and subsequent sepsis. Moreover, in this study we chose to administer DcR3 Fc 30 min before and 6 h after CLP. The protective effect of DcR3 Fc in CLP mice might therefore be due to reduced inflammatory responses in the early phase and decreased lymphocyte apoptosis in the later phase.

One limitation of this study is that we only harvested blood and peritoneal lavage fluid after 24 h to detect the bacterial burden. The host immune response, rather than the pathogen, is primarily responsible for the morbidity and mortality associated with sepsis. DcR3 is a potent anti-inflammatory and anti-apoptotic factor that could activate inflammation-resolution programs and enhance host defenses to remove microorganisms. However, because DcR3 can inhibit apoptosis of infected cells, intracellular pathogens may survive and cause chronic infection. Further studies should extend the observation time past 24 h to evaluate the effects of DcR3 on CLP-induced mice, as well as to examine the efficacy of DcR3 in patients in the clinical setting.

Taken together, our results suggest that DcR3 may have a vital role in the balance of pro-inflammatory and anti-inflammatory responses during sepsis. Because the available therapeutic strategies for sepsis are limited, DcR3 alone or in combination with antibiotics may be beneficial for patients with sepsis.

## References

[1] Angus DC, Linde-Zwirble WT, Lidicker J, Clermont G, Carcillo J, Pinsky MR. Epidemiology of severe sepsis in the United States: analysis of incidence, outcome, and associated costs of care. Crit Care Med. 2001;29:1303–1310. 11445675 10.1097/00003246-200107000-00002

[2] Martin GS, Mannino DM, Eaton S, Moss M. The epidemiology of sepsis in the United States from 1979 through 2000. N Engl J Med. 2003;348:1546–1554. 12700374 10.1056/NEJMoa022139

[3] Bone RC. The pathogenesis of sepsis. Ann Intern Med. 1991; 115:457–64. 1872494 10.7326/0003-4819-115-6-457

[4] Skrupky LP, Kerby PW, Hotchkiss RS. Advances in the management of sepsis and the understanding of key immunologic defects. Anesthesiology. 2011;115:1349–62. 10.1097/ALN.0b013e31823422e8 21952252 PMC3433833

[5] Cinel I, Dellinger RP. Advances in pathogenesis and management of sepsis. Curr Opin Infect Dis. 2007;20:345–9. 17609592 10.1097/QCO.0b013e32818be70a

[6] Ward PA. Immunosuppression in sepsis. JAMA. 2011;306:2618–21. 10.1001/jama.2011.1831 22187286

[7] Exline MC, Justiniano S, Hollyfield JL, Berhe F, Besecker BY, Das S, et al Microvesicular caspase-1 mediates lymphocyte apoptosis in sepsis. PLoS One. 2014;9:e90968 10.1371/journal.pone.0090968 24643116 PMC3958341

[8] Harjai M, Bogra J, Kohli M, Pant AB. Is suppression of apoptosis a new therapeutic target in sepsis? Anaesth Intensive Care 2013;41:175–83. 23530784 10.1177/0310057X1304100207

[9] Yan Z, Li J, Lou J, Zhou Y, Bo L, Zhu J, et al Upregulation of programmed death-1 on T cells and programmed death ligand-1 on monocytes in septic shock patients. Critical Care. 2011;15:R70–9 10.1186/cc10059 21349174 PMC3222003

[10] Ayala A, Perl M, Venet F, Lomas-Nerira J, Swan R, Chunq CS. Apoptosis in sepsis: mechanisms, clinical impact and potential therapeutic targets. Curr Pharm. 2008;14:1853–59. 10.2174/138161208784980617 18691096

[11] Manjuck J, Saha DC, Astiz M, Eales LJ, Rackow EC. Decreased response to recall antigens is associated with depressed costimulatory receptor expression in septic critically ill patients. J Lab Clin Med. 2000;135:153–60. 10695660 10.1067/mlc.2000.104306

[12] Nolan A, Weiden M, Kelly A, Hoshino Y, Hoshino S, Mehta N, et al CD40 and CD80/86 act synergistically to regulatate inflammation and mortality in polymicrobial sepsis. Am J Respir Crit Care Med. 2008;177:301–08. 17989345 10.1164/rccm.200703-515OC PMC2218847

[13] Toda M, Kawamot T, Ueha T, Kishimoto K, Hara H, Fukase N, et al 'Decoy' and 'non-decoy' functions of DcR3 promote malignant potential in human malignant fibrous histiocytoma cells. Int J Oncol. 2013;43:703–12. 10.3892/ijo.2013.1999 23817777 PMC3787885

[14] Chen L, Tian X, Li W, Agarwal A, Zhuang G. Expressions of Fas/DcR3 and RGD-FasL mediated apoptosis in pituitary adenomas. Neurol India. 2009;57:28–30. 19305072 10.4103/0028-3886.48808

[15] Havashi S, Miura Y, Nishiyama T, Mitani M, Tateishi K, Sakai Y, et al Decoy receptor 3 expressed in rheumatoid synovial fibroblasts protects the cells against Fas-induce apoptosis. Arthritis Rheum. 2007;56:1067–75. 17393415 10.1002/art.22494

[16] Lin WW, Hsieh SL. Decoy receptor 3: a pleiotropic immunomodulator and biomarker for inflammatory disease, autoimmune disease and cancer. Biochem Pharmacol. 2011;81:838–47. 10.1016/j.bcp.2011.01.011 21295012

[17] Lee CS, Hu CY, Tsai HF, Wu CS, Hsieh SL, Liu LC, et al Elevated serum decoy receptor 3 with enhanced T cell activation in systemic lupus erythematosus. Clin Exp Immunol. 2008;151:383–90. 10.1111/j.1365-2249.2007.03579.x 18190609 PMC2276966

[18] Hou YQ, Xu P, Zhang M, Han D, Peng L, Liang DY, et al Serum decoy receptor 3, a potential new biomarker for sepsis. Clin Chim Acta. 2012;413:744–8. 10.1016/j.cca.2012.01.007 22280900

[19] Hubbard WJ, Choudhry M, Schwacha MG, Kerby JD, Rue LW, Bland KI, et al Cecal ligation and puncture. Shock. 2005;24:52–7. 16374373 10.1097/01.shk.0000191414.94461.7e

[20] Xie K, Yu Y, Pei Y, Hou L, Chen S, Xiong L, et al Protective effects of hydrogen gas on murine polymicrobial sepsis via reducing oxidative stress and HMGB1 release. Shock. 2010;34:90–7,. 10.1097/SHK.0b013e3181cdc4ae 19997046

[21] Macher GS, Aulmann S, Wagener N, Funke B, Taqscherer KE, Haferkamp A, et al Decoy receptor 3 is a prognostic factor in renal cell cancer. Neoplasia. 2008;10:1049–56. 18813347 10.1593/neo.08626 PMC2546583

[22] Tu HF, Liu CJ, Liu SY, Chen YP, Yu EH, Lin SH, et al Serum decoy receptor 3 level: a predictive marker for nodal metastasis and survival among oral cavity cancer patients. Head Neck. 2011;33:396–402. 10.1002/hed.21467 20645287

[23] Chen CY, Yang KY, Chen MY, Chen HY, Lin MT, Lee YC, et al Decoy receptor 3 levels in peripheral blood predict outcomes of acute respiratory distress syndrome. Am J Respir Crit Care Med. 2009;180:751–60. 10.1164/rccm.200902-0222OC 19644047

[24] Cardinale CJ, Wei Z, Panossian S, Wang F, Kim CE, Mentch FD, et al Targeted resequencing identifies defective variants of decoy receptor 3 in pediatric-onset inflammatory bowel disease. Genes Immun. 2013;14:447–52. 10.1038/gene.2013.43 23965943

[25] Hsu TL, Wu YY, Chang YC, Yang CY, Lai MZ, Su WB, et al Attenuation of Th1 response in decoy receptor 3 transgenic mice. J Immunol. 2005;175:5135–45. 16210617 10.4049/jimmunol.175.8.5135

[26] Herzum I, Renz H. Inflammatory markers in SIRS, sepsis and septic shock. Curr Med Chem 2008;15:581–7. 18336272 10.2174/092986708783769704

[27] Pierrakos C, Vincent JL. Sepsis biomarkers: a review. Crit Care 2010;14:R15–33. 10.1186/cc8872 20144219 PMC2875530

[28] van den Berg JW, van der Zee M, de Bruin RW, van Holten-Neelen J, Naqtzaam NM, IJzermans JN, et al Mild vs. strong anti-inflammatory therapy during early sepsis in mice: a matter of life and death. Crit Care Med. 2011;39:1275–81. 10.1097/CCM.0b013e31820edf75 21336123

[29] Panacek EA, Marshall JC, Albertson TE, Johnson DH, Johnson S, MacArthur RD, et al Efficacy and safety of the monoclonal anti-tumor necrosis factor antibody F(ab’)2 fragment afelimomab in patients with severe sepsis and elevated interleukin-6 levels. Crit Care Med. 2004;32:2173–82. 15640628 10.1097/01.ccm.0000145229.59014.6c

[30] Boomer JS, To K, Chang KC, Takasu O, Osborne DF, Walton AH, et al Immunosuppression in patients who die of sepsis and multiple organ failure. JAMA. 2011;306:2594–605. 10.1001/jama.2011.1829 22187279 PMC3361243

[31] Hotchkiss RS, Swanson PE, Knudson CM, Chang KC, Cobb JP, Osborne DF, et al Overexpression of Bcl-2 in transgenic mice decreases apoptosis and improves survival in sepsis. J Immunol. 1999;162:4148–56. 10201940

[32] Matsuda N, Yamamoto S, Takano K, Kaqeyama S, Kurobe Y, Yoshihara Y, et al Silencing of fas associated death domain protects mice from septic lung inflammation and apoptosis. Am J Respir Crit Care Med. 2009;179:806–15. 10.1164/rccm.200804-534OC 19201926

[33] Yun Z, Tao ZH, Ye Tian, Zhu J, Cao L, Deng X, et al Ginsenoside Rg1 improves survival in a murine model of polymicrobial sepsis by suppressing the inflammatory response and apoptosis of lymphocytes. Journal of Surgical Research. 2013;183:760–766. 10.1016/j.jss.2013.01.068 23478085

[34] Funke B, Autschbach F, Kim S, Lastitschka F, Strauch U, Roqler G, et al Functional characterisation of decoy receptor 3 in Crohn's disease. Gut. 2009; 58:483–91. 10.1136/gut.2008.148908 19039087

[35] Ayala A, Xu YX, Ayala CA, Sonefeld DE, Karr SM, Evans TA, et al Increased mucosal B-lymphocyte apoptosis during polymicrobial sepsis is a Fas ligand but not an endotoxinmediated process. Blood. 1998;91:1362–72. 9454767

[36] Papathanassoglou EDE, Moynihan JA, McDermott MP, Ackerman MH. Expression of Fas (CD95) and Fas ligand on peripheral blood mononuclear cells in critical illness and association with multiorgan dysfunction severity and survival. Crit Care Med. 2001;29;709–18. 11373453 10.1097/00003246-200104000-00002

